# Case of Softening of the Heart in a Person Who Was Believed to Have Died of Starvation and Exhaustion

**Published:** 1847-07

**Authors:** B. G. Darley

**Affiliations:** Coolock


*Case of Softening of the Heart in a Person who was believed to have died of Starvation and Exhaustion.* By B. G. Darley, M. D. Coolock.—On the 4th of January, 1847, I was requested by the coroner to examine the body of an elderly female, who was reported to have died of want and starvation in this neighborhood. The history of the case was shortly this: she was a poor woman, who obtained her living by wandering about from place to place, and was in the habit occasionally of stopping a day or two in the house in which she died. She had come there three days before the above date, and complained of much weakness, and was suffering, as the people in the cabin said, from "a kind of asthma." She had some tea to drink, but eat nothing. She died on the 3rd instant, apparently from exhaustion. On opening the chest, the lungs appeared healthy, and collapsed slightly; there was no water in the pericardium, the heart was larger than natural, the auricles greatly distended and full of blood. On lifting up the apex to see the size of the heart, my fingers went through the substance of the left auricle, and this with a very slight pressure indeed; out of the rent made by my finger poured a great quantity of fluid blood which filled the pericardium; the right auricle was in the same condition, literally choked with fluid blood, and in this auricle it was of a very dark colour; but the most remarkable character was the softened state of the walls of both auricles, particularly the left; they were of the same colour and as friable as the liver, and not unlike portions of lung affected with pulmonary apoplexy. The increase size of the heart appeared chiefly made up by the great dilatation and distention of the auricles; the ventricals did not appear larger than natural; they were empty of blood, and their muscular structure was of a pale colour.

The viscera of the abdomen were generally healthy; the stomach was contracted, and nothing in it but a half pint of a dark colored fluid; the intestines in parts were occupied by the same. The omentum was destitute of fat; indeed the absence of adipose tissue throughout the whole body was remarkable.

The brain was examined, and was perfectly healthy, but particularly bloodless.

Now what was the cause of death in this case? There was no lesion of the brains, lungs, or viscera of the abdomen, and though the heart was as described, it had preserved its integrity, at least there was no solution of continuity within its walls; and this might readily have taken place, considering the softened state of the auricular tissue, had the individual lived a little longer, and the auricles had power to act on their contents; in such case death, most probably, should have been laid to the door of a diseased heart, and not as, in my opinion, the result of an altered state of the contained blood.

The coroner's jury returned a verdict, that "death was caused by want and destitution."

In a physiological point of view, I should say that insufficiency of nutritious food rendered the heart unable to expel its contents, its muscular structure, particularly that of its auricular portions, was so softened and weakened as to allow of dilatation to the greatest possible amount; the blood gradually accumulating, congestion took place, and the woman died of what we may call congestive apoplexy of the auricles of the heart. The manner this is caused by starvation is thus: the blood is rendered thin, has little or no fibrin in it; the heart, along with the general muscular system, is weakened, and unable to expel its contents; congestion takes place; its cavities, particularly the auricles, yield to pressure; and, as is the case in all muscular cavities when distended beyond a certain extent, atony supervenes, the muscular fibres no longer contract, and death is the result.

From this examination, the difficulty of breathing during life may be explained, and, had an opportunity been afforded before death, we should probably have found the pulse slow and intermitting.

This case differs from the fatty degenerations of the heart in many particulars. First, this, as we have seen, engages the auricular portions of the heart, while it is the ventricles that are generally occupied by fatty deposition; again, it is in the corpulent and the full habit that the heart is predisposed to the fatty degeneration, whilst the softened auricle will be found in the ill-fed and destitute; again, the mode of death in the former has more of an apoplectic character, whilst in the latter the spark of life ebbs out from want of sustenance and vital power. In the fatty heart, the solids are the first to suffer, whilst in the other the mischief begins in impoverishment of the fluids.

As I fear the profession in this country will have many opportunities of examining the bodies of individuals dying under similar circumstances, though the case above noticed might have occurred at any period, I think it might be interesting for medical men to give reports of their examinations, and to observe the state of the several viscera, and especially the heart, in such cases.—*Ibid.*

